# Histone deacetylase 1 controls cardiomyocyte proliferation during embryonic heart development and cardiac regeneration in zebrafish

**DOI:** 10.1371/journal.pgen.1009890

**Published:** 2021-11-01

**Authors:** Anja Bühler, Bernd M. Gahr, Deung-Dae Park, Alberto Bertozzi, Alena Boos, Mohankrishna Dalvoy, Alexander Pott, Franz Oswald, Rhett A. Kovall, Bernhard Kühn, Gilbert Weidinger, Wolfgang Rottbauer, Steffen Just

**Affiliations:** 1 Molecular Cardiology, Department of Internal Medicine II, University of Ulm, Ulm, Germany; 2 Institute of Biochemistry and Molecular Biology, University of Ulm, Ulm, Germany; 3 Department of Internal Medicine II, University of Ulm, Ulm, Germany; 4 Department of Internal Medicine I, University of Ulm, Ulm, Germany; 5 Department of Molecular Genetics, Biochemistry and Microbiology, University of Cincinnati College of Medicine, Cincinnati, Ohio, United States of America; 6 Department of Pediatrics, University of Pittsburgh, and Richard King Mellon Institute for Pediatric Research and Division of Pediatric Cardiology, Children’s Hospital of Pittsburgh of UPMC, Pittsburgh, Pennsylvania, United States of America; University of North Carolina, UNITED STATES

## Abstract

In contrast to mammals, the zebrafish maintains its cardiomyocyte proliferation capacity throughout adulthood. However, neither the molecular mechanisms that orchestrate the proliferation of cardiomyocytes during developmental heart growth nor in the context of regeneration in the adult are sufficiently defined yet. We identified in a forward genetic N-ethyl-N-nitrosourea (ENU) mutagenesis screen the recessive, embryonic-lethal zebrafish mutant *baldrian* (*bal*), which shows severely impaired developmental heart growth due to diminished cardiomyocyte proliferation. By positional cloning, we identified a missense mutation in the zebrafish *histone deacetylase 1* (*hdac1*) gene leading to severe protein instability and the loss of Hdac1 function *in vivo*. Hdac1 inhibition significantly reduces cardiomyocyte proliferation, indicating a role of Hdac1 during developmental heart growth in zebrafish. To evaluate whether developmental and regenerative Hdac1-associated mechanisms of cardiomyocyte proliferation are conserved, we analyzed regenerative cardiomyocyte proliferation after Hdac1 inhibition at the wound border zone in cryoinjured adult zebrafish hearts and we found that Hdac1 is also essential to orchestrate regenerative cardiomyocyte proliferation in the adult vertebrate heart. In summary, our findings suggest an important and conserved role of Histone deacetylase 1 (Hdac1) in developmental and adult regenerative cardiomyocyte proliferation in the vertebrate heart.

## Author summary

Heart disease is one of the most common causes of death in all developed countries. While zebrafish cardiomyocytes are able to proliferate throughout adulthood, mammalian cardiomyocytes lose this ability during early development, and therefore are not capable to replace and renew cardiomyocytes after injury. The underlying mechanisms of cardiomyocyte proliferation are still not completely resolved. Understanding how zebrafish cardiomyocytes preserve their proliferating state, would be a valuable information to foster cardiac regeneration, e.g. after myocardial infarction in patients. Knowledge of the signaling pathways that need to be activated, or deactivated in order to induce cardiomyocyte proliferation after acute or chronic injury will pave the way for the development of genetic and/or pharmacological treatment options. In an ENU-mutagenesis screen, we identified the zebrafish mutant *baldrian*, which shows reduced embryonic cardiomyocyte proliferation. As genetic cause of the observed phenotype, we identified a missense mutation in the *hdac1* gene. By treatment of heart-injured adult fish with the HDAC1 inhibitor Mocetinostat, we were able to show a reduced rate of cardiomyocyte proliferation also in the adult zebrafish heart *in vivo*, suggesting a role of Hdac1 in embryonic heart growth and adult regenerative cardiomyocyte proliferation in zebrafish.

## Introduction

The adult human heart is unable to sufficiently regenerate after myocardial infarction. For decades, adult mammalian cardiomyocytes were thought to be post-mitotic due to irreversible cell cycle withdrawal. Only recently, low level cardiomyocyte proliferation and turnover was described in adult mammalian hearts, which unfortunately is insufficient to regenerate the heart after acute or chronic damage [1,2]. Studies in animal models which show a high cardiac regenerative capacity such as zebrafish implied that mechanisms driving cardiomyocyte proliferation during development might also be key to stimulate postnatal regenerative cardiomyocyte proliferation in the mammalian heart. The control of cardiomyocyte proliferation in the developing heart depends on both, cell cycle activating and inhibiting pathways, and both mechanisms also seem to play a fundamental role in the induction of postnatal cardiomyocyte proliferation. For instance, Tbox transcription factor 20 (Tbx20) was found to play a crucial role in the control of cardiomyocyte proliferation in the developing heart in zebrafish and mice [3–5], but also in the adult heart since transgenic induction of Tbx20 in adult mice significantly augments cardiomyocyte proliferation after myocardial injury by inducing proliferative and repressing cell cycle inhibitory pathways [6].

Histone modifications are known to be crucial players in the epigenetic regulation of heart development [7,8]. Deacetylation of histone tails by histone deacetylases (HDACs) usually promotes chromatin condensation and thereby repression of gene transcription. HDAC1 and 2, both class I HDACs, are known to be ubiquitously expressed but to play essential roles during cardiac development in mice [9–11]. Simultaneous cardiac-specific deletion of HDAC1 and 2 results in the development of severe cardiac defects such as dilated cardiomyopathy and arrhythmias which lead to early neonatal lethality. Cardiac-specific knockout of either murine HDAC1 or HDAC2 alone does not interfere with cardiac development or function since HDAC1 and 2 are functionally redundant and compensate each other reciprocally. By contrast, global knockout of only HDAC1 in mice leads to lethality before embryonic day 10.5 due to severe developmental abnormalities and proliferation defects [11]. Very recently, Song et al. demonstrated in zebrafish that Hdac1 also plays an important role in controlling outflow tract development by the regulation of the proliferation of second heart field (SHF) progenitors [12].

In order to identify novel regulators of embryonic heart growth, we defined here the genetic underpinnings of the ethylnitrosourea (ENU)-induced recessive-lethal zebrafish mutant *baldrian* (*bal*^*hc050*^). Homozygous *baldrian* mutants display severely reduced cardiomyocyte numbers in the embryonic ventricle due to a missense mutation within the zebrafish *histone deacetylase 1 (hdac1)* gene. The *bal* mutation results in destabilization and degradation of Hdac1 and consequently leads to reduction of histone deacetylation in *baldrian* mutant embryos. Furthermore, we show here that Hdac1 controls both cardiomyocyte proliferation during embryonic heart growth and after cardiac injury in adult zebrafish.

## Materials and methods

### Ethics statement

All procedures and experiments in this study were carried out after appropriate institutional approvals (Tierforschungszentrum (TFZ) Ulm University, No. 0183 and Regierungspräsidium Tübingen No. 1415), which conform to the EU Directive 2010/63/EU.

### Animals

Care and breeding of zebrafish *Danio rerio* was carried out as described [13]. Unless otherwise stated, the AB wildtype strain was used for injection experiments. Adult *baldrian (bal)* mutants were kept as heterozygous fish, breeding resulted in 25% homozygous *bal* mutant offspring. Pictures and movies were recorded at the 18 somite stage and at 48, 72 and 96 hours post fertilization (hpf). For documentation, zebrafish embryos were treated with 0.003% 1-phenyl-2-thiourea to inhibit pigmentation. We established the cardiomyocyte-specific reporter line *Tg(myl7*:*NLS-mCherry)* expressing a N-terminally NLS-tagged mCherry under control of the *myl7* (*myosin light polypeptide 7*) promotor [14,15]. For regeneration experiments the *Tg(myl7*:*NLS-mCherry)* line, as well as the double transgenic line *Tg(fli*:*GFP*^*y1Tg*^;*myl7*:*NLS-mCherry* were used, marking cardiomyocytes (CMs) with nuclear mCherry expression and endothelial cells with GFP expression.

### Microinjection of fertilized zebrafish oocytes

Morpholino-modified antisense oligonucleotides (MOs; Gene Tools, LLC, Oregon, USA) were injected into one-cell stage zebrafish embryos. To knock-down zebrafish *hdac1*, MO targeting the translational start site of zebrafish *hdac1* (MO-*hdac1*: 5´-TTGTTCCTTGAGAACTCAGCGCCAT-3´) or standard control MO (MO-*control*: 5´-CCTCTTACCTCAGTTACAATTTATA -3´) were injected into fertilized oozytes at the one-cell stage. MOs were injected with 6.1 ng in 0.2 M potassium chloride.

Zebrafish *hdac1* mRNA was prepared using the mMessage mMachine SP6 Transcription Kit (Invitrogen AM1340). 100 ng/μl *hdac1* mRNA in 0.2 M potassium chloride was injected in the fertilized oocyte before the first cell cleavage.

### Pharmacological treatment

Mocetinostat (Selleckchem #S1122) and Parthenolide (Selleckechem #S2341) were dissolved in DMSO (Sigma #D2650). Mocetinostat reveals most potent inhibitory activity against HDAC1, but also inhibits in a dose dependent manner other class I HDAC enzymes such as HDAC2. Zebrafish embryos were treated with 10 μM (Mocetinostat in E3) or 7.5 μM (Parthenolide in E3) for 72 h from 0 hpf, the respective amount of DMSO was used as control. The inhibitor solution was refreshed every 24 hours.

### Protein lysate extraction and Western blot analysis

Protein extraction of embryonic zebrafish and immunoblotting was carried out as described [16,17]. For immunoblotting following primary antibodies were used: rabbit anti-Hdac1 (1:500, Abcam #ab33278), rabbit anti-acetyl-Histone H3 (1:1000, Millipore #06–599) rabbit anti-acetyl Histone H4 (1:1000, Abcam #ab177790). For loading control rabbit anti-Histone H3 (1:2500,Sigma/ #H0164), rabbit anti-Pan Cadherin (1:10000, Abcam #ab16505), or rabbit anti-LaminB1 (1:1000; Abcam #16048) were used. Signals were detected by chemiluminescence (anti-mouse IgG HRP-linked, anti-rabbit IgG HRP-linked, Cell Signaling #7076/#7074) using a Luminescent image analyzer (Image Quant LAS4000mini, D-79111 Freiburg, Germany).

### Cryoinjury of adult zebrafish hearts, drug treatment and dissection of embryonic hearts

Cryoinjury of adult zebrafish hearts were performed as described before [18]. Cryoinjured fish were incubated in fish water containing the HDAC1 inhibitor Mocetinostat (Selleckchem #S1122, 5 μM) from 1–30 dpi (days post injury) in an incubator set at 27°C. DMSO treatment served as control. Solutions were changed every day. The dissection and IF of embryonic hearts at 72 hpf were carried out as described elsewhere [16].

### Immunofluorescence, histological staining and quantifications

Sectioning and immunostainings were performed as previously described [18]. Cardiomyocytes were identified via immunofluorescence against mCherry in the *Tg(myl7*:*NLS-mCherry)* transgenic line. The vascular system was analyzed via native GFP fluorescence in the *Tg(fli*:*GFP*^*y1*^*)* transgenic line. Primary antibodies used were: PCNA, Dako #M0879 (1:1000) and DsRed, Clontech #632496 (1:300). For PCNA staining, antigen retrieval was performed by incubating slides containing heart cryosections in 10 mM sodium citrate buffer (pH 6) for 10 min at 85°C. Secondary antibodies conjugated to Alexa 555 or 633 (Invitrogen) were used at a dilution of 1:1000. Nuclei were stained by DAPI (4′,6-diamidino-2-phenylindole, Sigma Aldrich #32670). Images of immunofluorescence stainings are single optical planes acquired at 20X magnification with a Leica Sp5 confocal microscope or with a Zeiss AxioObserver 7 microscope. For quantification of cardiomyocyte proliferation and wound revascularization, 2 sections displaying substantial wound as well as border myocardial tissue were analyzed per heart. Quantifications of PCNA positivity were performed in cardiomyocytes situated within 150 μm from the wound border. Acid fuchsin orange G (AFOG) staining was performed as previously described [19]. Measurements of the size of wound area and ventricle area on slides stained with AFOG were performed manually with ImageJ software on all sections of one serial slide, representing approximately one-sixth of the total ventricle.

### EdU incorporation and TUNEL assay in zebrafish embryos

For EdU incorporation assays in zebrafish embryos the Click-iT EdU Alexa Fluor488 Imaging Kit was used (Thermo Fisher #C10337). Therefore, embryos at 72 hpf were pulsed for 1h with EdU on ice afterwards hearts were dissected and stained for EdU, CM nuclei were stained with a rabbit anti-Mef2ac antibody (Santa Cruz # sc-313). TUNEL assays were performed using DeadEnd Fluoro metric TUNEL System (Promega G3250) on dissected zebrafish hearts. For experiments using the transgenic line *Tg(myl7*:*NLS-mCherry)* hearts were dissected and stained for EdU after a 1.5 h pulse with EdU on ice.

### Histology and Whole mount in situ hybridization

For histology, fixed embryos were embedded in JB-4 (Polysciences, Inc.,Warrington, PA, USA) and 5 μm sections were cut, dried and stained with hematoxylin & eosin [20].

In situ hybridization was conducted as described elsewhere [21]. The original protocol was modified by replacing the 24-well plate by placing the embryos in 1.5ml Eppendorf tubes. Probes against *cmlc1* and *vmhc* were used.

### Imaging and statistical analysis

Whole mount zebrafish images were taken with an Olympus SZX 16 microscope. To count cardiomyocytes on dissected hearts, the hearts were stained and mounted. Z-stack images were taken with either the iMIC Microscope with a step size of 1μm and a 20x air objective or the Leica DMi8 confocal microscope with a 63x oil objective. Counting of CM number was performed with the ImageJ cell counter plugin. For statistics, the data was analyzed using GraphPad Prism6. All results are expressed as means ± standard derivation (s.d.) and analyses were performed using Mann-Whitney U or two tailed t-test, a value of P<0.05 was accepted as statistically significant.

## Results

### The *baldrian* mutation interferes with embryonic cardiomyocyte proliferation in zebrafish

**Fig 1:**
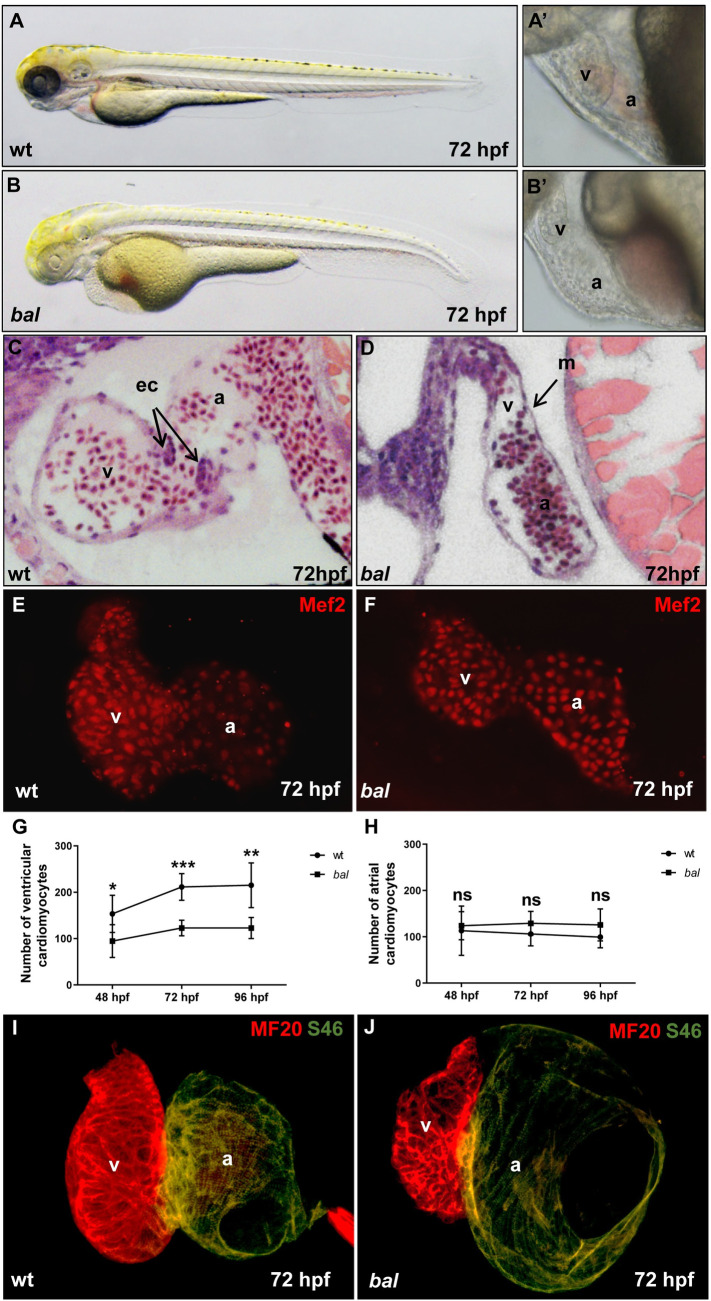
The *baldrian* mutation interferes with embryonic cardiomyocyte proliferation in zebrafish. (A-B) Lateral view of 72 hpf wild-type sibling (wt) (A) and *bal* embryo (B). (A’, B’) Close-up of the heart region of wt (A’) and *bal* mutants (B’) displayed small ventricle of *bal* mutants. (C, D) Histological sections showed normal morphology of the wt heart (C) and a monolayered myocardium and small ventricle in the *bal* mutant heart. M, myocardium; ec, endocardial cushions; v, ventricle; a, atrium. (E, F) Embryonic zebrafish hearts stained with antibodies against MEF-2 to visualize cardiomyocyte nuclei. Cardiomyocyte (CM) numbers appear reduced in *bal* mutants compared to wild-type siblings. (G) Ventricular cardiomyocyte numbers at 48, 72 and 96 hpf, were significantly decreased in *bal* (48 hpf wt: 153.37 ± 39.91, n = 7 and *bal*: 94.71± 35.22, n = 8, p = 0.014; 72 hpf wt: 211.42 ± 28.81, n = 7 and *bal*: 122.85 ± 16.88, n = 7, p = 0.001; 96 hpf wt: 215.20 ± 48.35, n = 10 and *bal* 122.83 ± 22.83, n = 6, p = 0.001). Error bars indicate s.d., *p < 0.05, **p < 0.01, ***p < 0.001, ns, not significant. (H) Atrial CM numbers were unaltered (48 hpf p = 0.332, 72 hpf p = 0.135, 96 hpf p = 0.147). (I, J) Immunofluorescent (IF) staining against meromyosin (MF20) (red) and atrial-specific myosin (S46) (green) demonstrated normal chamber specification of *bal* hearts.

In order to identify novel key-regulators of embryonic heart growth, we characterized here the ENU-induced recessive, embryonic-lethal zebrafish mutant *baldrian* (*bal*^*hc050*^) (Tübingen 2000). Homozygous *bal* mutants display a small cardiac ventricular chamber at 72 hours post fertilization (hpf), whereas the size of the atrium appears normal (Fig 1A, 1B, 1A´ and 1B´). Histological analyses at 72 hpf revealed that myocardial and endocardial layers are defined in *bal* mutant ventricles, however, the *bal* ventricular myocardium remains thin, whereas in wild-types the myocardial cell layer regularly thickens by the addition of cardiomyocytes (Fig 1C and 1D). To assess cardiomyocyte numbers in *bal* mutant hearts, we dissected hearts at three developmental time points, 48, 72 and 96 hpf, and stained these hearts with antibodies against MEF-2 (Myocyte Enhancer Factor 2) to specifically label all cardiomyocyte nuclei (Fig 1E and 1F). At 48, 72 and 96 hpf, we found significantly less cardiomyocytes in *bal* ventricular chambers compared to wild-type siblings (48 hpf wt: 153.37 ± 39.91, n = 7 and *bal*: 94.71± 35.22, n = 8, p = 0.014; 72 hpf wt: 211.42 ± 28.81, n = 7 and *bal*: 122.85 ± 16.88, n = 7, p = 0.001; 96 hpf wt: 215.20 ± 48.35, n = 10 and *bal* 122.83 ± 22.83; n = 6, p = 0.002) (Fig 1G). The number of atrial cardiomyocytes was not reduced in *bal* mutants (48 hpf wt: 113.12 ± 53.29, n = 8and *bal*: 123.85 ± 30.24, n = 7, p = 0.332; 72 hpf sib: 106.00 ± 25.90; n = 7 and *bal*: 129.14 ± 25.84; n = 7, p = 0.135; 96 hpf wt: 99.40 ± 23.17; n = 10 and *bal*: 125.50 ± 34.59 n = 6, p = 0.147) (Fig 1H), suggesting a heart growth defect specific to the ventricular chamber. Differentiation and specification of cardiomyocytes was not impaired as revealed by antibody staining of the heart chamber specific myosin heavy chains, with S46 antibody labelling the atrium and MF20 labeling both atrium and ventricle (Fig 1I and 1J), indicating that altered cardiomyocyte specification is not the reason for compromised proliferation.

**Fig 2:**
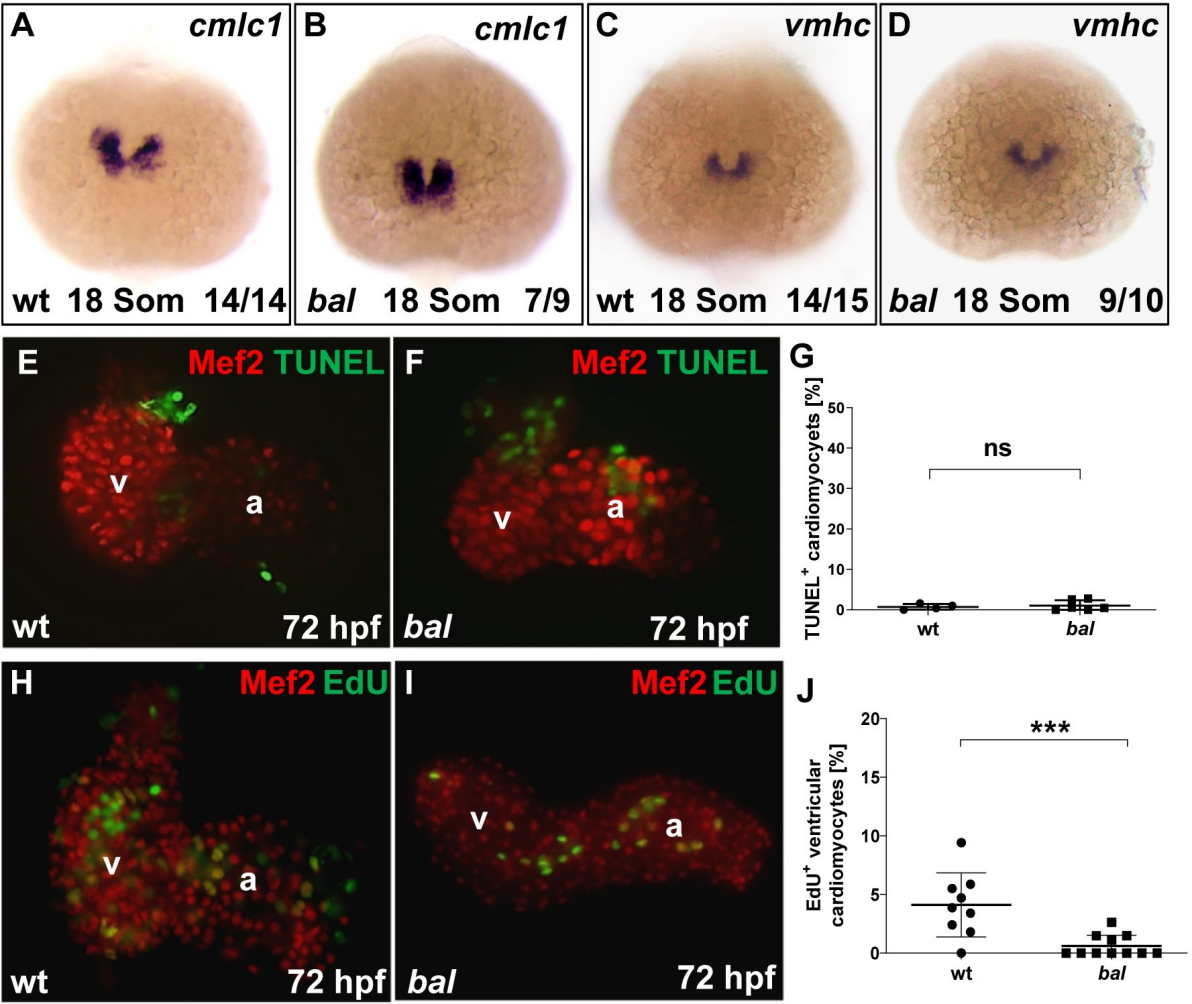
Early progenitor and chamber specification proceed normally in *bal* mutants. (A-D) Whole-mount antisense RNA in situ hybridization (ISH) of *cmlc1* and *vmhc* showed similar expression in *bal* mutants and wt siblings. (E, F) TUNEL staining (green) and IF for Mef2 (red) on dissected wt (E) and *bal* mutant hearts (F). (G) Quantification of TUNEL^+^ CMs revealed no alterations in apoptosis (wt 0.75 ± 0.70%, n = 4, bal 1.07 ± 1.28%, n = 6, p = 0.92), error bars indicate s.d., ns, not significant. (H, I) IF against Mef2 (red) and EdU (green) on dissected wt and *bal* hearts showed decreased numbers of EdU^+^ ventricular CMs. (J) Quantification revealed significantly decreased levels of EdU^+^ ventricular CMs in *bal* mutant hearts (wt 4.10 ± 2.73, n = 9, *bal* 0.61 ± 0.92, n = 11, p = 0.001). For significance testing Mann-Whitney-U test was applied, error bars indicate s.d., ***p < 0.001, ns, not significant.

Next, we assessed whether hypoplastic ventricular chambers in *bal* mutant embryos are caused by reduced amounts of cardiac precursors or by pathologically elevated cardiomyocyte apoptosis. To do so, we first conducted whole-mount antisense RNA in situ hybridization using probes detecting *cardiac myosin light chain 1 (cmlc1*, differentiating into atrial and ventricular cardiomyocytes*)* and *ventricular myosin heavy chain (vmhc*, differentiating into ventricle-specific cardiomyocytes*)* transcripts in the cardiac precursor cell population at the 18 somites stage. These analyses revealed no alterations in the number of cardiac precursor cells that differentiate into atrial and ventricular cardiomyocytes between *bal* mutants and wild-type siblings (Fig 2A–2D). Additionally, we performed TUNEL assays on dissected embryonic hearts at 72 hpf to visualize cardiomyocyte apoptosis and found no significant increase in apoptotic cardiomyocyte numbers in *bal* mutant hearts compared to wild-type siblings (wt: 1.07 ± 1.28%, n = 6 and *bal*: 0.75 ± 0.70% n = 4; p = 0.92) (Fig 2E–2G). These findings imply that not accelerated apoptosis but rather diminished proliferation might be the molecular cause of hypoplasia in embryonic *bal* mutant hearts.

Hence, to assess whether proliferation of ventricular cardiomyocytes is indeed impaired in *bal*, we performed EdU-stainings to mark cells in the S-phase of the cell cycle combined with MEF-2 immunostainings at 72 hpf (Fig 2H and 2I). We found EdU^+^ (proliferating) ventricular cardiomyocytes significantly reduced in *bal* mutants compared to wild-type siblings (wt: 5.78 ± 3.93, n = 9 and *bal*: 0.55 ± 0.93, n = 11) (S1 Fig). By calculating the mitotic index of *bal* mutant and wild-type hearts, we found a significant reduction of cardiomyocyte proliferation (Mitotic index: wt: 4.10 ± 2.72% n = 9 and *bal*: 0.61 ± 0.91%n = 11; p = 0.001) (Fig 2J), demonstrating that impaired proliferation of ventricular cardiomyocytes is the sole molecular cause for cardiac hypoplasia in *bal* mutants.

### *Baldrian* (*hc050*) locus encodes zebrafish *histone deacetylase 1* (*hdac1*)

**Fig 3:**
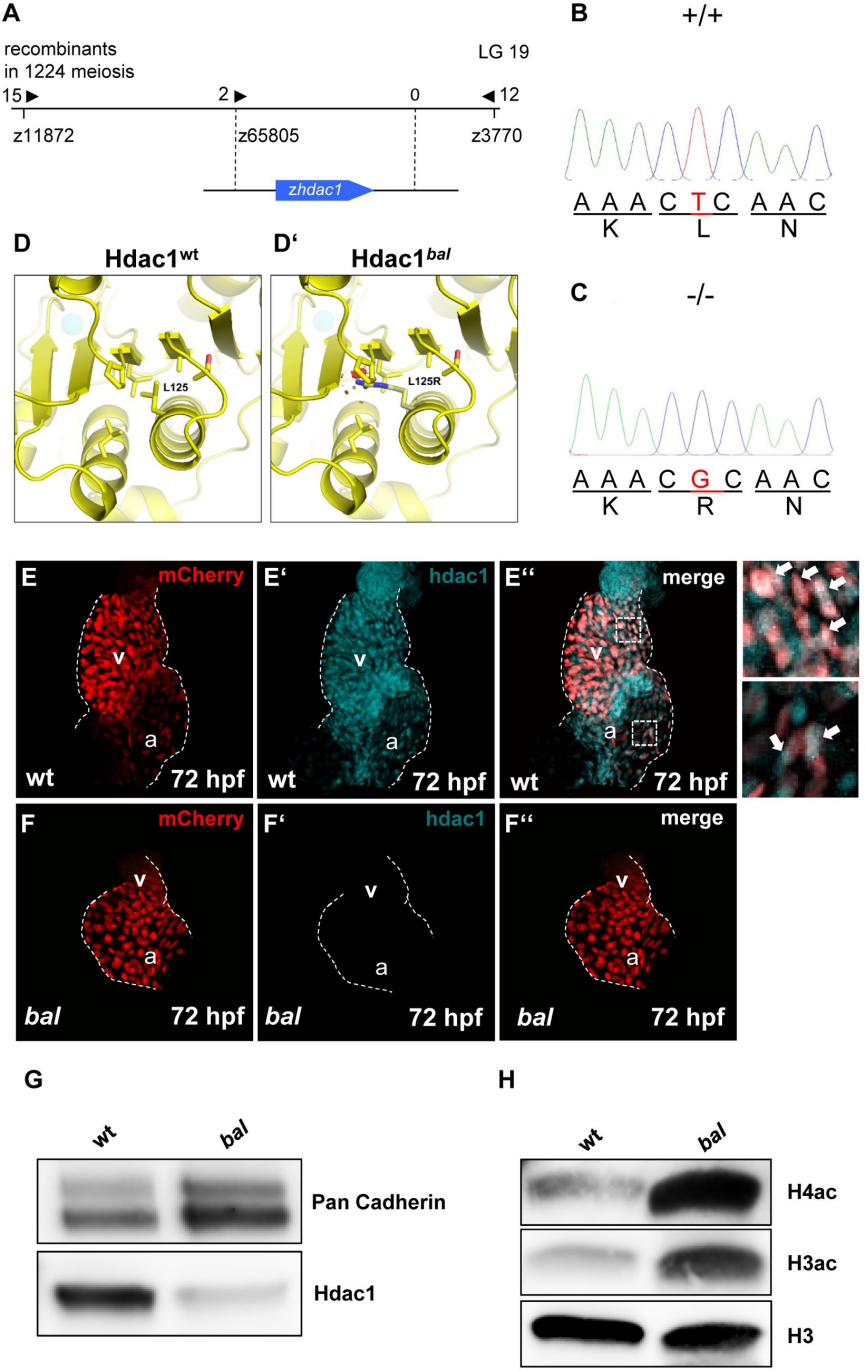
*Bal* phenotype is caused by a point mutation in the Hdac1 gene. (A) Genetic map of the *baldrian*/Hdac1 gene locus. The *bal* mutation interval is flanked by the microsatellite markers z11872, z65805 and z3770. (B, C) Sequencing revealed the *bal* mutation to be a thymidine-to-guanine nucleotide transition in codon 125 within the zebrafish *hdac1* gene. (D, D’) Homology model for *Danio rerio* Hdac1 (drHdac1), AAH85375, built from human HDAC1 structure 4BKX. wt and *bal* mutant structure of drHdac1, highlighting the active site zinc ion (cyan sphere) and L125. (D) Zoomed view of L125, showing the nonpolar interactions it makes with neighboring side chains within the hydrophobic core of drHDAC1. (D’) Modelled L125R mutant Hdac1. Steric clashes are represented as red disks. Modeled Arg rotamer was chosen based on the least number of steric clashes. (E-F”) IF staining on dissected hearts at 72 hpf, Hdac1 (turquoise) of *Tg(myl7*:*mCherry)* hearts (E-E”) or *bal;Tg(myl7*:*mCherry)* mutant hearts (F-F”) white arrows show Hdac1 costained cardiomyocytes of wt ventricle or atrium. (G) Western blot analysis of Hdac1 levels on protein lysates from *bal* and wt, demonstrating a drastic reduction in Hdac1 protein level in the *bal* mutants. (H) Western blot analysis of acetylation levels of Histone 3 (H3ac) and 4 (H4ac) of *bal* and wt protein lysates show a pronounced hyperacetylation of H3 and H4 in *bal* mutant embryos. pan Histone 3 served as loading control.

To identify the ENU-induced mutation that causes the recessive *bal* ventricular hypoplasia phenotype, we performed a genome-wide study of microsatellite marker segregation by bulked segregant analysis. We mapped *bal* to zebrafish chromosome 19 in-between the microsatellite markers z65805 and z3770. Recombination analysis of 612 *bal* mutant embryos and genetic fine-mapping restricted the *bal* to a genomic interval including the zebrafish homologue of the human *histone deacetylase 1* (*hdac1*) gene (Fig 3A). Next, to define the ENU-induced *bal* mutation, we sequenced the entire coding sequence of zebrafish *hdac1* from wild-type and *bal* mutant cDNA and genomic DNA. We identified the *bal* mutation to be a thymidine-to-guanine nucleotide transition in codon 125 of the zebrafish *hdac1* gene, predicted to change the amino acid leucine at position 125 to an arginine (L125R) (Fig 3B and 3C). Zebrafish Hdac1 consists of 480 amino acids and presents 90.2% amino acid identity to human Hdac1 (S2A Fig). The identified missense mutation (L125R) resides within the highly conserved deacetylase domain of Hdac1.

To investigate whether the identified missense mutation in Hdac1 results in structure perturbing alterations of the mutant protein, we next used the human HDAC1 structure (pdb 4BKX) (S2B Fig) [22], SWISS-MODEL, and the PyMol software for *in silico* homology modeling of both, zebrafish Hdac1^wt^ and Hdac1^L125R^ proteins (Fig 3D). L125 of zebrafish Hdac1 is mostly buried and points towards the hydrophobic core of the protein (Fig 3D). The L125R mutation induces steric clashes with P295 and L296 (Fig 3D). Therefore, folding of Hdac1^L125R^ is predicted to be seriously impaired and mutant HDAC1 protein destabilized.

To validate the *in silico* prediction that the identified *baldrian hdac1* mutation likely results in the destabilization of Hdac1 proteins, we performed immunostainings using a HDAC1-specific antibody. We found Hdac1 severely diminished in *bal* mutant hearts at 72 hpf (Fig 3E–3F”). To validate this result, we conducted Western blot analyses and found that Hdac1 protein levels in *bal* mutant protein lysates were barely detectable in *bal* mutants (Fig 3G). These findings prove that the identified Hdac1^L125R^ mutation, as predicted by our *in silico* modeling, significantly interferes with Hdac1 protein stability *in vivo*.

Next, to assess whether loss of Hdac1 in zebrafish interferes with overall histone deacetylation in *bal* mutant embryos, we performed Western blot analyses using antibodies against acetylated Histone 4 (H4ac) and acetylated Histone 3 (H3ac). We found H3 and H4 acetylation severely upregulated in *bal* mutant embryos (Fig 3H), demonstrating that loss of Hdac1 in *bal* interfered with regular histone deacetylation *in vivo*.

**Fig 4:**
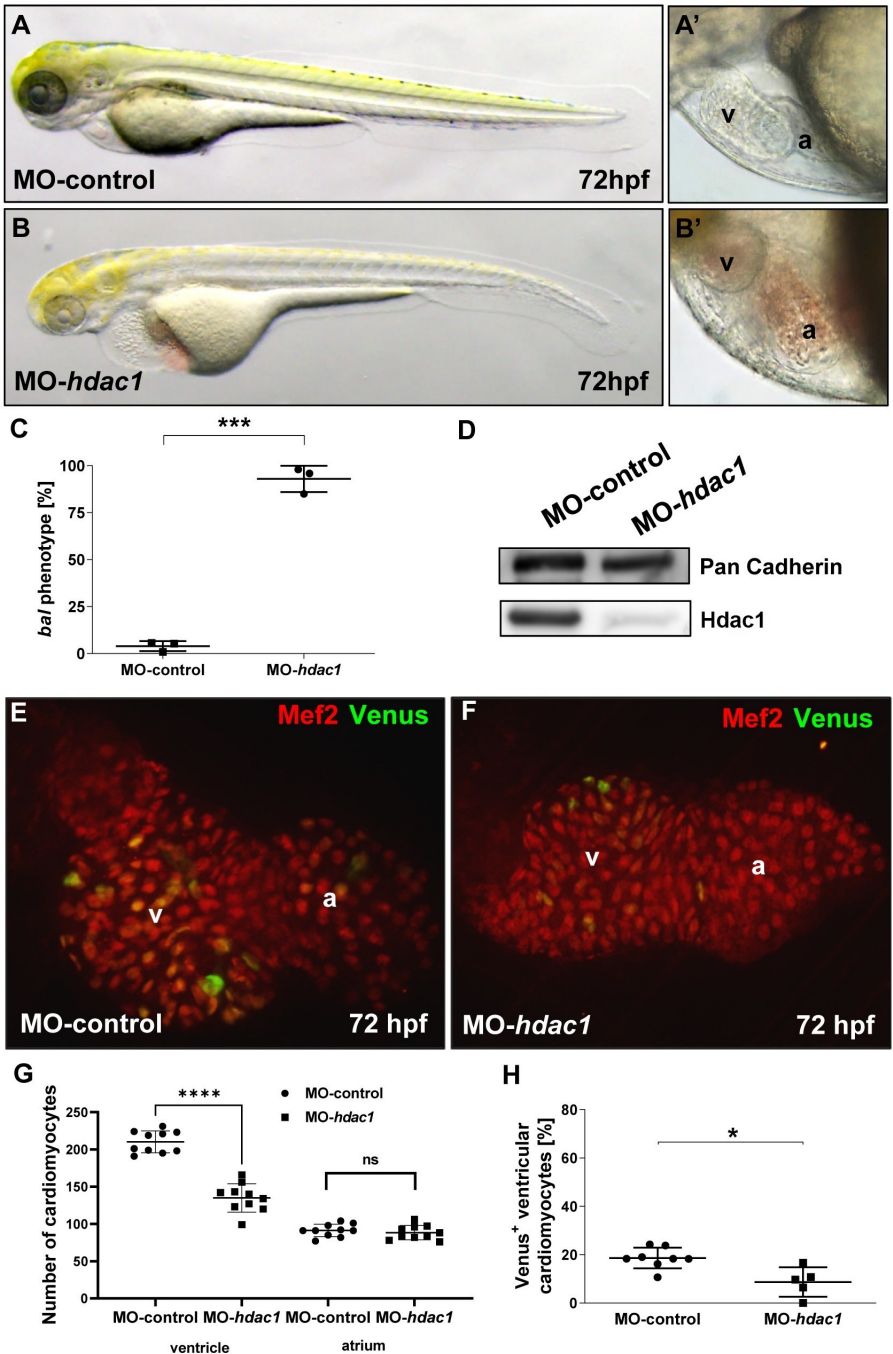
Morpholino-mediated knockdown of *hdac1* phenocopies *bal* mutants. (A-B’) Lateral view of MO-control (A, A’) and MO-*hdac1* (B, B’) at 72 hpf, injection of MO-*hdac1* led to the *bal* heart phenotype (B’). (C) Statistical analysis of MO-*hdac1* injection. MO-*hdac1* injection highly significantly (p = 0.0001) induced the *bal* phenotype (245/264) compared to Ctrl-MO (10/248). Significance was calculated using two-tailed t-test, error bars show s.d., asterisks indicate significance. (D) Western blot analysis of lysates either from MO-*hdac1* or MO-control injected embryos depicting reduced Hdac1 protein levels in the morphants. (E, F) IF staining for Mef2 (red) and Venus (green) on dissected hearts from embryos either injected with MO-*hdac1* (E) or MO-control (F). (G, H) Statistical analysis of CM number (G) and cycling CMs (H) demonstrated a reduced CM number in the ventricle (MO-control 210.2 ± 14.68, MO-hdac1 135,0 ± 19.06, n = 10, p < 0.0001) but not in the atrium (MO-control 91.20 ± 8.38, MO-*hdac1* 88.2 ± 9.57, n = 10, p = 0.2079). Furthermore, a decreased cycling rate (p = 0.01) was revealed in MO-*hdac1* injected embryos (H). Error bars indicate s.d.; *p < 0.05, ****p < 0.0001, ns, not significant.

To substantiate our finding that impaired embryonic cardiomyocyte proliferation in *bal* mutant zebrafish is indeed due to loss of Hdac1 function, we next injected Morpholino-modified antisense oligonucleotides directed against the translational start site of zebrafish *hdac1* into one-cell-stage wild-type zebrafish embryos (Fig 4A and 4B). We found that 92.93 ± 6.99% of MO injected embryos (N = 264 injected embryos in n = 3 independent experiments; p = 0.0001) (Fig 4C) displayed the *bal* “small heart” phenotype at 72 hpf (Fig 4B’). We also evaluated cardiomyocyte proliferation in MO-*hdac1* injected embryos using the transgenic cell cycle reporter line *Tg(myl7*:*Venus-hGeminin*^*pd58*^*)* expressing Venus during S/G2/M phase, cardiomyocytes were counterstained with Mef2 antibody [23] (Fig 4E and 4F). Similar to the situation in *bal* mutant hearts, embryonic cardiomyocyte proliferation was significantly reduced in MO-*hdac1*-injected zebrafish embryos at 72 hpf, leading to a reduction of ventricular cardiomyocyte numbers in *hdac1* morphant hearts (MO-Control 210.2 ± 14.68, n = 10 and MO-*hdac1* 135.0 ± 19.06, n = 10, p < 0.0001) but unchanged cardiomyocyte number in the atrium (MO-control 91.20 ± 8.38, n = 10, MO-*hdac1* 88.2 ± 9.57, n = 10, p = 0.2079) (Fig 4G). As a measurement of ventricular cardiomyocyte cycling the percentage of Venus^+^ cardiomyocytes in the ventricle was calculated and a reduction in Venus^+^ cardiomyocytes was found in *hdac1* morphant hearts (18.62 ± 4.27%, n = 8 in MO-control and 8.72 ± 6.10%, n = 5, p = 0.01 in MO-*hdac1*) (Fig 4H).

**Fig 5:**
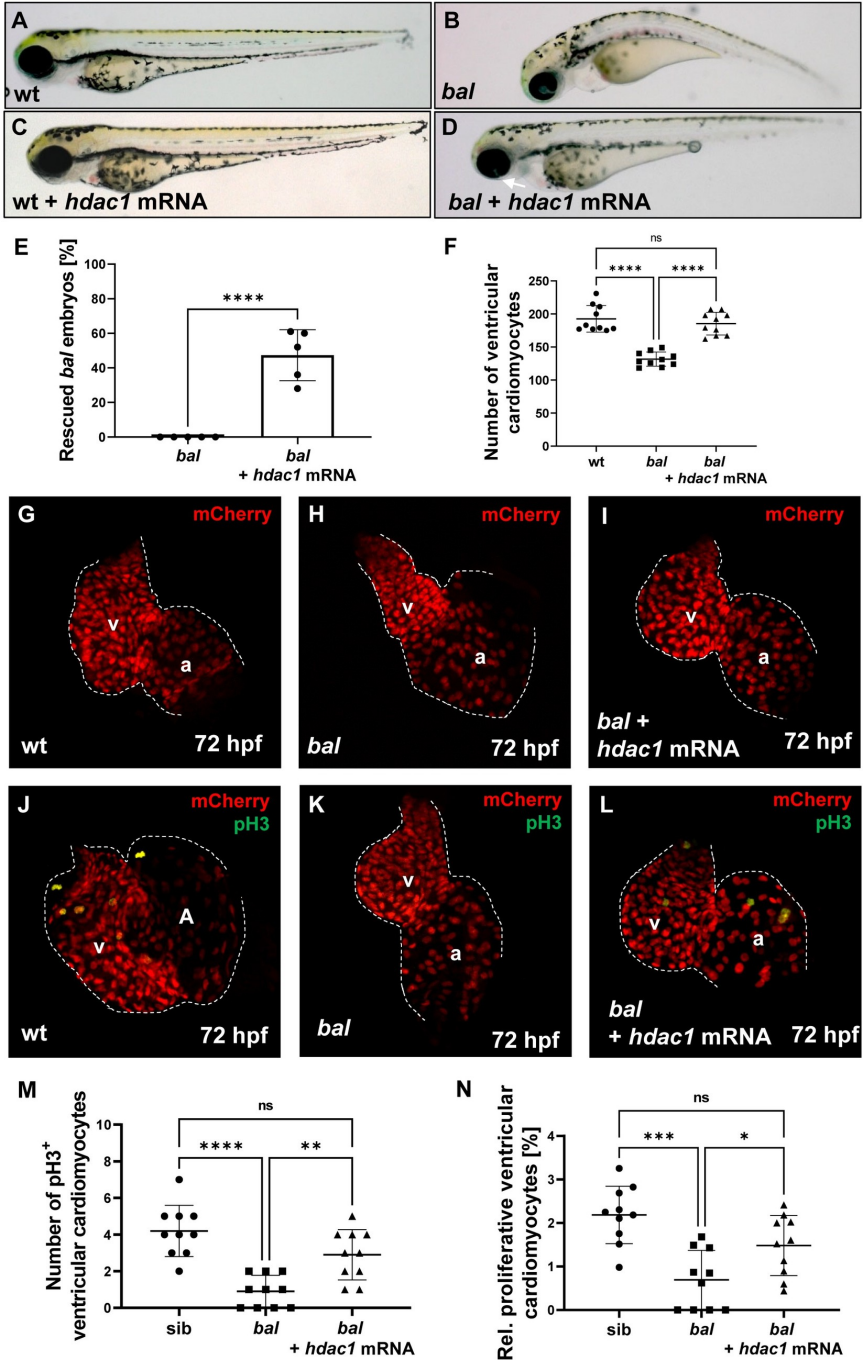
*Hdac1* mRNA injection rescues the *bal* phenotype. (A-E) Lateral view of uninjected and *hdac1* mRNA injected wildtyp (A, C) or *bal* embryos (B, D) at 72 hpf, injection of mRNA prevents the *bal* phenotype in mutant embryos (uninjected 0 ± 0%, *hdac1* mRNA injected 47.4 ± 14.76%, n = 50 embryos). (F-I) Cardiomyocytes (red) in *Tg(myl7*:*mCherry)*, *bal*;*Tg(myl7*:*mCherry)* and *hdac1* mRNA injected *bal*;*Tg(myl7*:*mCherry)* hearts. *Hdac1* mRNA injection prevents the loss of ventricular cardiomyocytes in *bal* mutant embryos. (J-N) Statistical analysis of pH3^+^ cardiomyocytes after *hdac1* mRNA injection in *bal*;*Tg(myl7*:*mCherry)* mutant embyos (wt: 4.2 ± 1.40; *bal*: 0.9 ± 0.88; *bal* + *hdac1* mRNA: 2.9 ± 1.37, n = 10) and the relative proliferation rate (wt: 2.19 ± 0.66%; *bal*: 0.70 ± 0.68%; *bal* + *hdac1* mRNA: 1.48 ± 0.69%, n = 10). Error bars indicate s.d.; *p < 0.05, **p < 0.01, ***p < 0.001, ****p < 0.0001, ns, not significant.

To verify that loss of Hdac1 function leads to the observed phenotype, we performed a rescue experiment using *hdac1* mRNA. The injection of *hdac1* mRNA prevented the characteristic phenotype in *bal* mutant embryos in almost half of the embryos (47.4 ± 14.76%, n = 50) (Fig 5A–5E). Also, the number of ventricular cardiomyocytes was significantly higher compared to *bal* mutant embryos (wt: 192.6 ± 20.16, n = 10; *bal*: 131.6 ± 10.64, n = 10; *bal* + *hdac1* mRNA: 185.3 ± 17.15, n = 10) (Fig 5F). The rate of proliferating pH3^+^ cardiomyocytes was increased in *hdac1* mRNA injected mutant embryos, compared to uninjected mutants (wt: 2.19 ± 0.66%, n = 10; *bal*: 0.70 ± 0.68%, n = 10; *bal* + *hdac1* mRNA: 1.48 ± 0.69%, n = 10) (Fig 5G–5N).

Our findings strongly suggest that the *bal* mutant phenotype is the result of histone deacetylase 1 (Hdac1) loss-of-function.

### Pharmacological inhibition of Hdac1 in zebrafish embryos mimics the *bal* phenotype

**Fig 6:**
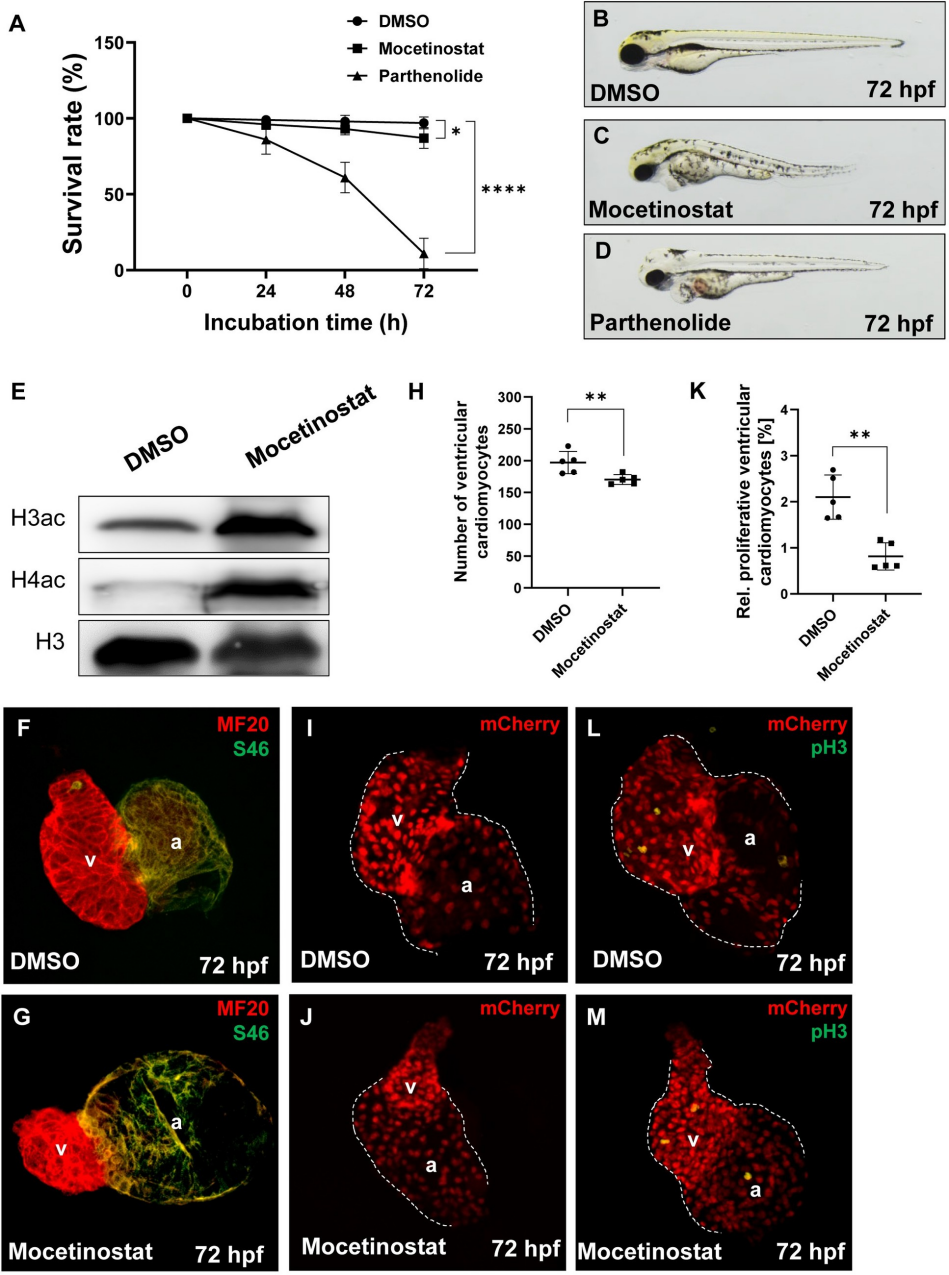
Mocetinostat treatment of zebrafish embryos induces the *bal* phenotype. (A) Survival rate of Mocetinostat- and Parthenolide-treated embryos, compared to the DMSO control. At 24 hpf (DMSO: 99.0 ± 2.0%; Mocetinostat: 96.0 ± 3.27%; Parthenolide: 86.0 ± 9.52%, n = 4), at 48 hpf (DMSO: 98.0 ± 4.0%; Mocetinostat: 93.0 ± 3.83%; Parthenolide: 61.0 ± 10.0%, n = 4) and at 72 hpf (DMSO: 97.0 ± 3.83%; Mocetinostat: 87.0 ± 6.83%; Parthenolide: 11.0 ± 10.0%, n = 4). (B-D) Lateral view of inhibitor- or DMSO-treated embryos at 72 hpf. (E) Western blot analysis of acetylation levels of Histone 3 (H3ac) and 4 (H4ac) of lystes from Mocetinostat and DMSO-treated embryos, show a pronounced hyperacetylation of H3 and H4 in Mocetinostat-treated embryos. pan Histone 3 served as a loading control. (F-G) IF staining against meromyosin (MF20) (red) and atrial-specific myosion (S46) (green) demonstrated normal chamber specification of Mocetinostat-treated hearts. (H-J) Number of ventricular cardiomyocytes was reduced in Mocetinostat treated *Tg(myl7*:*mCherry)* embryos (DMSO: 197.0 ± 17.39; Mocetinostat: 170.4 ± 7.7, n = 5). (K-M) The rate of ventricular pH3^+^ cardiomyocytes was reduced in Mocetinostat-treated embryos as well (DMSO: 2.1 ± 0.48%; Mocetinostat: 0.8± 0.30%, n = 5). Error bars indicate s.d., *p < 0.05, **p < 0.01, ****p < 0.0001.

To assess the impact of pharmacological HDAC1 inhibition on cardiomyocyte proliferation *in vivo*, two different Hdac1 inhibitors were tested on wild-type zebrafish embryos. Incubation of embryos with 10 μM Mocetinostat resulted in a phenotype comparable with that observed in *bal* mutants. Parthenolide another HDAC1 inhibitor, had a very high mortality rate already at a concentration of 7.5 μM but did not show a phenotype at lower concentrations (24 hpf: DMSO 99.0 ± 2.0%, n = 40; Mocetinostat 96.0 ± 3.27%, n = 40; Parthenolide 86.0 ± 9.52%, n = 40, 48 hpf: DMSO 98.0 ± 4.0%; Mocetinostat 93.0 ± 3.83%; Parthenolide 61.0 ± 10.0%, 72 hpf: DMSO 97.0 ± 3.83%; Mocetinostat 87.0 ± 6.83%; Parthenolide 11.0 ± 10.0%) (Fig 6A–6D). Western blot analysis of whole embryo lysates of Mocetinostat-treated embryos showed an increase in Histone 3 and Histone 4 acetylation, as observed in *bal* mutants (Fig 6E). Differentiation of the two heart chambers, visualized by MF20 and S46 immunostaining was unaffected by Mocetinostat treatment (Fig 6F and 6G). The number of ventricular cardiomyocytes (DMSO: 197.0 ± 17.39, n = 5; Mocetinostat: 170.4 ± 7.7, n = 5) as well as the rate of pH3^+^ cardiomyocytes (DMSO: 2.1 ± 0.48%, n = 5; Mocetinostat: 0.8± 0.30%, n = 5) was significantly decreased in Mocetinostat-treated embryos compared to DMSO control embryos (Fig 6H–6M).

These findings support the hypothesis that Mocetinostat is a suitable and valid drug to assess the impact of Hdac1 inhibition in cardiomyocyte proliferation *in vivo*.

### Hdac1 inhibition blocks cardiomyocyte proliferation, but not revascularization or scar resorption during heart regeneration in adult zebrafish

**Fig 7:**
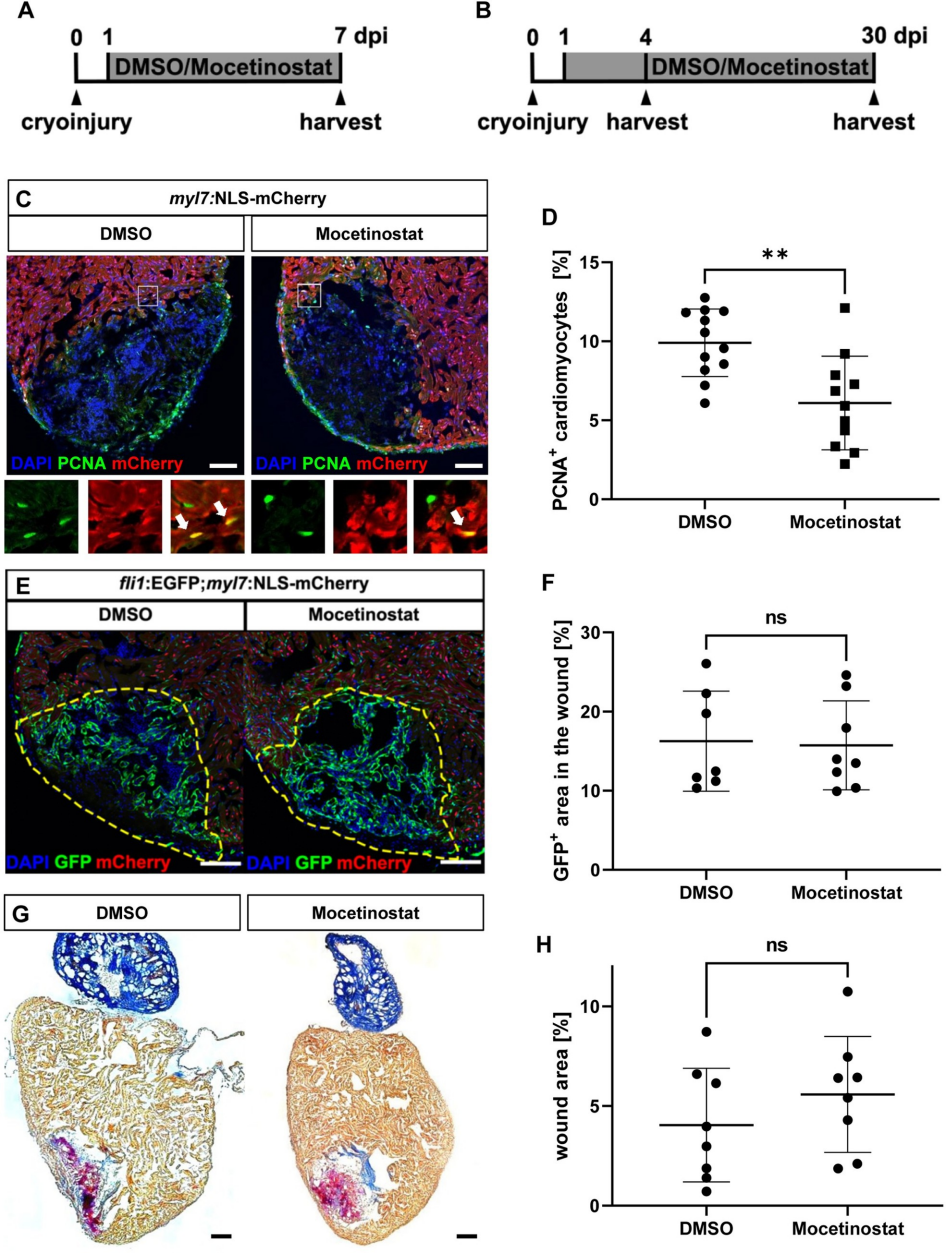
Hdac1 is involved in heart regeneration after cryoinjury. (A, B) Experimental timeline of cryoinjury, treatment and harvesting of the hearts for the analysis of cardiomyocyte proliferation (A) revascularization (4 dpi) and scar formation (30 dpi) (B). (C, D) Immunohistochemistry for mCherry (red, cardiomyocytes) and PCNA (green) on 7 dpi hearts of *Tg(myl7*:*NLS-mCherry)* fish treated with either DMSO or Mocetinostat from 1 to 7 dpi, showed reduced rate of cycling cardiomyocytes at the wound border zone in Mocetinostat-treated hearts (% of PCNA^+^/mCherry^+^ cardiomyocytes DMSO 9.91 ± 2.14, n = 12, Mocetinostat 6.1 ± 2.96, n = 11, p = 0.0018). (E, F) Native GFP (green, endothelial cells) and mCherry (red, cardiomyocytes) fluorescence in 4 dpi hearts of *Tg(fli*:*GFP*^*y1*^*;myl7*:*NLS-mCherry)* fish treated with DMSO or Mocetinostat from 12 hpi to 4 dpi, showed no changes in revascularization (% of GFP^+^ wound area: DMSO Ctrl 16.26 ± 6.34, n = 7, Mocetinostat 15.74 ± 5.62, n = 8, p > 0.9999). (G, H) Acid Fuchsin Orange G (AFOG) staining of 30 dpi hearts treated with DMSO or Mocetinostat from 1 to 30 dpi, have no significant difference in wound size (DMSO 4.05 ± 4.85, n = 8, Mocetinostat 5.59 ± 2.91, n = 8, p = 0.3282). Error bars indicate s.d.; **p < 0.01, ns, not significant. Scale bars, 100 μm.

In contrast to mammals, adult zebrafish are able to structurally and functionally regenerate their hearts in response to injury [24,25]. Zebrafish cardiac regeneration relies mainly on cell cycle re-entry of spared cardiomyocytes at the wound border [24,26]. Another important feature of zebrafish cardiac repair is that scarring is transient, since the ECM deposited in the wounded area within the first days post cryoinjury is gradually resorbed until little or no scar tissue remains in the regenerated heart [25,27–29]. Restoration of the healthy myocardium is supported also by revascularization of the injured area, an early response that starts at 15 hours post injury (hpi) with the formation of the first coronary vessel sprouts from the wound border and is completed by 4 days post injury (dpi) before the onset of cardiomyocyte proliferation [30]. Since homozygous *bal* mutants do not survive to adulthood, we utilized Mocetinostat as Hdac1 inhibitor to assess whether Hdac1 activity is required for regeneration of the adult zebrafish heart after cryoinjury. Hearts were collected for analysis at different days post injury to measure the effect of Hdac1 inhibition on distinct aspects of regeneration, namely cardiomyocyte proliferation (7 dpi, Fig 7A), revascularization (4 dpi, Fig 7B) and scar resorption (30 dpi, Fig 7B). To assess whether incubation with Mocetinostat in the fish water changes the acetylation state of histones as observed in Mocetinostat-treated embryos, we incubated fish with 0.5 μM Mocetinostat for 5 days. Western blot of dissected ventricles showed highly elevated levels of Histone 3 and Histone 4 acetylation compared to the DMSO control (S3 Fig).

First, to assess cardiomyocyte cell cycle re-entry upon Hdac1 inhibition, we measured PCNA positivity in mCherry^+^ border zone cardiomyocytes of 7 dpi *Tg(myl7*:*NLS-mCherry)* fish treated from 1 to 7 dpi (Fig 7A). We found that cardiomyocyte proliferation rate was ~40% lower in Mocetinostat-treated fish compared to DMSO-treated control (% of PCNA^+^/mCherry^+^ cardiomyocytes DMSO 9.91 ± 2.14, n = 12 Mocetinostat 6.1 ± 2.96, n = 11, p = 0.0018) (Fig 7C and 7D), demonstrating that, similar to the situation in HDAC1-deficient embryonic zebrafish hearts, HDAC1 inhibition also leads to decreased cardiomyocyte proliferation in adult regenerating zebrafish hearts at 7 dpi. Next, to investigate whether Hdac1 regulates revascularization, injured *Tg(fli*:*GFP*^*y1*^;*myl7*:*NLS-mCherry)* fish were treated with Mocetinostat from 12 hpi to 4 dpi (Fig 7B). The extent of the coronary plexus formed by endothelial cells at 4 dpi, measured as percentage of GFP^+^ area inside the mCherry^-^ injured area, was unaltered by Hdac1 inhibition (DMSO 16.26 ± 6.34%, n = 7, Mocetinostat 15.74 ± 5.62%, n = 8, p > 0.9999) (Fig 7E and 7F). Scar resorption was analyzed by Acid Fuchsin Orange G (AFOG) staining on hearts treated from 1 to 30 dpi (Fig 7A), a time point when typically cryoinjured hearts show only a fraction of the initial scar left [25,27,29]. Interestingly, the size of the remaining wound was only slightly, but not significantly bigger in the Mocetinostat-treated fish, compared to the DMSO control group at 30 dpi (DMSO 4.05 ± 4.85%, n = 8, Mocetinostat 5.59 ± 2.91%, n = 8, p = 0.3282) (Fig 7G and 7H), suggesting that, although cardiomyocyte proliferation was found decreased at the wound border zone at 7 dpi, injured hearts can, at least, initiate regenerative processes such as scar resorption or revascularization after Hdac1 inhibition. Future studies are needed to assess whether Hdac1-depleted, injured adult zebrafish hearts can ultimately regenerate and if yes, what mechanisms compensate for the loss of Hdac1 function during cardiac regeneration.

We conclude that Hdac1 inhibition, in addition to its impact on the proliferation of embryonic zebrafish cardiomyocytes, also interferes with cardiomyocyte proliferation in the regenerating adult zebrafish heart 7 days post cryoinjury.

## Discussion

The genetic and molecular mechanisms that drive embryonic heart growth are only insufficiently understood yet. Interestingly, some of the known signaling pathways and processes that control heart growth during development are already shown to play crucial roles also in the context of heart regeneration or repair after myocardial injury in the adult heart. During the past decade, the zebrafish evolved as an excellent model organism to define and study the mechanisms that orchestrate both, embryonic heart growth and the regeneration in the adult heart [2,24,31–33]. Here, we dissected the genetic underpinnings of the ENU-induced, embryonic-lethal zebrafish mutant *baldrian* which shows impaired embryonic heart growth due to a mutation in the *hdac1* gene. We demonstrated here for the first time that loss of Hdac1 interferes with (1) cardiomyocyte proliferation in the embryonic zebrafish heart and with (2) regenerative cardiomyocyte proliferation in the adult zebrafish heart after cryoinjury.

HDAC1, together with HDAC2, HDAC3 and HDAC8, is a member of the class I family of HDACs and particularly HDAC1 and HDAC2 share high sequence homology (85%). In mice, global knockout of HDAC1 resulted in early embryonic lethality at gastrulation (E10.5) [34]. By contrast, cardiac-specific deletion of HDAC1 does neither interfere with cardiac development nor heart function. Similarly, cardiac-specific deletion of HDAC2 has also no adverse effect on heart development or function, whereas cardiac-specific knockout of both, HDAC1 and HDAC2 together, results in severe cardiac defects including dilated cardiomyopathy and arrhythmias and thereby neonatal lethality [11], implying functional redundancy of HDAC1 and HDAC2 in mammals. Interestingly, zebrafish lack Hdac2, allowing the investigation of the exclusive functions of Hdac1 in the vertebrate heart *in vivo* without influencing and compensating effects by a Hdac2 ortholog. Very recently, Song and coworkers found by studying another zebrafish *hdac1* mutant allele that Hdac1 controls the development of the cardiac outflow tract (OFT) by inhibiting the expression of the retinoic acid-responsive gene *ripply3* in second heart field (SHF) progenitor cells [12]. We found here that Hdac1, in addition to its role in the SHF, controls embryonic heart growth by orchestrating cardiomyocyte proliferation at later stages during development. Lagger and coworkers found HDAC1 deficiency in mice to interfere with cell proliferation in diverse tissues and organs [34], leading to early embryonic lethality. Additionally, postnatal loss of HDAC1/2 in lung epithelium resulted in increased expression of potent cell-cycle regulators such as Rb1 or p21 interfering with regular cell-cycle progression and thereby cell proliferation [35]. An effect of HDAC1 deficiency on cell proliferation in the vertebrate heart was not demonstrated so far.

Loss of Hdac1 in *baldrian* zebrafish mutants leads to reduced ventricular cardiomyocyte numbers. As depicted by the analysis of DNA synthesis using EdU incorporation in *bal* mutant hearts, we found significantly impaired cardiomyocyte proliferation without affecting cardiomyocyte specification or cardiomyocyte apoptosis in Hdac1-deficient hearts. Using the established HDAC1 inhibitor Mocetinostat on zebrafish embryos, we found that proliferation of cardiomyocytes is significantly diminished by the loss of Hdac1 function. Histone deacetylases are known to catalyze the removal of acetyl groups from histone tails, thereby orchestrating the chromatin structure by compaction usually resulting in transcriptional repression [36]. Here, we found the massive hyperacetylation of histones in (1) *bal* mutant hearts, but also (2) in zebrafish embryos as well as adult zebrafish treated with the HDAC1 Inhibitor Mocetinostat, demonstrating on the one hand loss of Hdac1 function in these models and secondly a possible epigenetic effect on gene transcription. The role of epigenetic regulation of cardiomyocyte proliferation was underscored by several studies. Interestingly, global knockout of HDAC2 in mice results in massively increased proliferation of ventricular cardiomyocytes and perinatal lethality of the animals [10]. Although cardiomyocyte proliferation was found to be increased, the cause for this phenomenon does not seem to reside within cardiomyocytes since cardiomyocyte-specific deletion of HDAC2 does not result in this phenotype [11]. Furthermore, specific histone marks such as H3K9/14, H3K18 and H3K27 acetylation were found to be reduced in post-mitotic cardiomyocytes again underlining the importance of epigenetic regulatory mechanisms of cardiomyocyte proliferation [37].

Within the damaged heart key signaling pathways of embryonic heart development particularly pathways that are involved in the control of embryonic heart growth such as Notch, Wnt/ß-catenin, TGFβ/BMP or Tbx, are often re-activated [38–40]. In this context, it was recently shown that experimental activation of developmental pathways such as BMP2 and Tbx20 can foster cardiomyocyte survival and proliferation in the uninjured and injured adult mouse heart [6,41,42]. Whether epigenetic regulatory mechanisms such as histone modifications or epigenetic regulators also contribute to this phenomenon is largely unknown.

In contrast to mammals, zebrafish retain the high ability to fully regenerate their hearts after injury throughout adulthood. Genetic fate-mapping experiments demonstrated that after acute cardiac damage, spared cardiomyocytes de-differentiate, re-enter the cell-cycle, and proliferate to replace the myocardial tissue [25,32,43,44]. Recent research has begun to identify molecular regulators of zebrafish heart regeneration [26]. In this context, several signaling pathways including fibroblast growth factor, retinoic acid, transforming growth factor β (TGF-β), insulin-like growth factor, Jak1/Stat3, Notch, Bone Morphogenetic Protein (BMP), and Neuregulin signaling [18,45,46] were found to play important roles during naturally occurring heart regeneration in zebrafish. We show here for the first time that Hdac1 function is crucial for the regenerative proliferation of cardiomyocytes in the adult zebrafish heart. By cryoinjury of the adult zebrafish heart and the subsequent inhibition of Hdac1 starting 1 day after injury, we found significantly diminished cardiomyocyte proliferation at the wound border zone at 7 dpi. By contrast, Hdac1 inhibition did not interfere with the revascularization of the injured area, one of the critical processes that guarantee heart regeneration in zebrafish. Furthermore, by investigating scar size at 30 dpi, we found only a slightly increased scar area in Hdac1-ablated zebrafish hearts, suggesting that although the resorption of scar tissue might be less effective, cardiac regenerative processes including scar resorption appear to be initiated. Whether cardiac regeneration is complete and functional in Hdac1-deficient zebrafish hearts at 60–90 dpi needs to be investigated in future studies. Irrespective of the outcome of these analyses, we will either elucidate an essential role of Hdac1 during heart regeneration or we will be able to dissect pathways that enable to bypass or overcome the Hdac1-associated deficit in cardiomyocyte proliferation. In this context, the direct molecular consequences particularly transcriptional alterations caused by disturbed Hdac1-associated epigenetic regulations during embryonic heart growth and regenerative cardiomyocyte proliferation in the adult zebrafish need to be deciphered.

In summary, we identified here the epigenetic regulator Hdac1 as a molecular signal that regulates cardiomyocyte proliferation in the embryonic zebrafish heart. Additionally, Hdac1 controls cardiomyocyte proliferation in the adult zebrafish heart after myocardial injury, suggesting that Hdac1 might be a potential pro-regenerative target for stimulating regenerative processes also in the injured mammalian heart.

## Supporting information

S1 Fig *Bal* mutants display reduced numbers of EdU-positive ventricular CMs. (A) EdU incorporation assay showed significantly reduced numbers of EdU^+^ CMs in the ventricle of *bal* mutants at 72 hpf compared to their wt siblings (wt: 5.78 ± 3.93, n = 9 and *bal*: 0.55 ± 0.93, n = 11). (B-D) Statistical analysis of atrial cardiomyocyte numbers (wt: 97.29 ± 11.54; *bal*: 90.43 ± 7.48, n = 7), EdU+ cardiomyocytes in the atrium (wt: 3.71 ± 1.38, *bal*: 2.00 ± 1.53, n = 7) and the proliferative index in the atrium (wt: 3.79 ± 1,22%, *bal*: 2.22 ± 1.65%, n = 7). Error bars indicate s.d., ***p < 0.001, ns, not significant.
(TIF)

S2 Fig Zebrafish Hdac1 (zHdac1) and human HDAC1 (hHDAC1) are evolutionary highly conserved. (A) Amino acid (aa) alignment of zHdac1 and hHDAC1 depicts high amino acid identity across species. Identical aa in black, similar aa in grey and no similarity in white. The red box and the asterisk indicate the *bal* mutation at position 125 of zebrafish Hdac1. (B) Overall structure of human HDAC1 (PDB4BKX, [22]) highlighting its secondary structure elements as cartoon presentation. The zinc ion (Zn) in the active center is shown as grey sphere. The leucine residue at position124 is shown as stick presentation (blue).
(TIF)

S3 Fig Treatment of adult fish with Mocetinostat results in hyperacetylation of histone 3 and 4 in the adult ventricle. (A, B) Western blot analysis of acetylation levels of Histone 3 (H3ac) and 4 (H4ac) of lysates of ventricles derived from Mocetinostat- and DMSO-treated fish show a pronounced hyperacetylation of H3 and H4 in inhibitor treated ventricles. LaminB1 served as loading control.
(TIF)
