# Supplementary figures and images for: Histone deacetylase 1 controls cardiomyocyte proliferation during embryonic heart development and cardiac regeneration in zebrafish
Source: PLoS Genet. 2021 Nov 1;17(11):e1009890. doi: 10.1371/journal.pgen.1009890 (PMC8584950; doi:10.1371/journal.pgen.1009890)

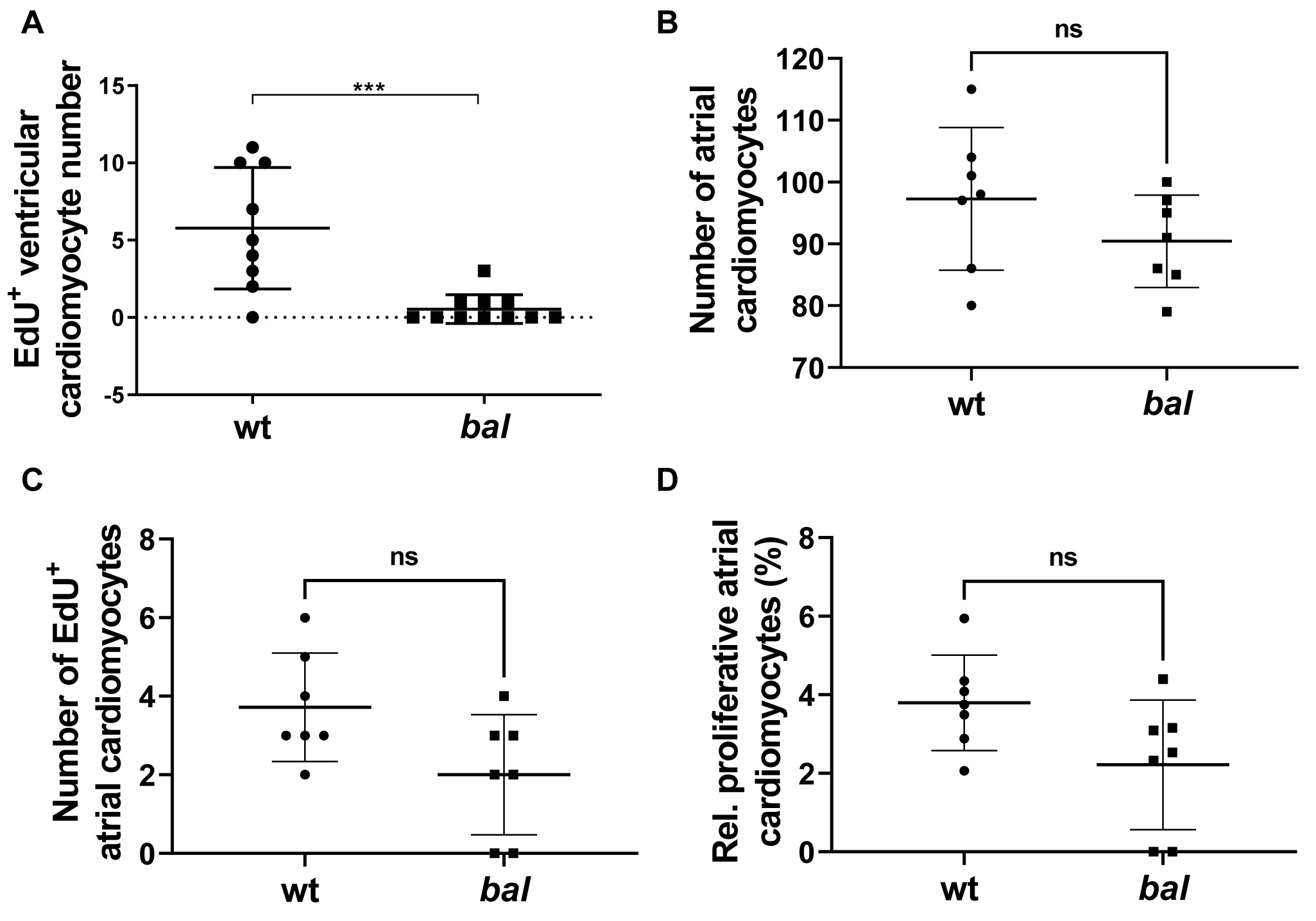

Supplement: S1 Fig — (A) EdU incorporation assay showed significantly reduced numbers of EdU+ CMs in the ventricle of bal mutants at 72 hpf compared to their wt siblings (wt: 5.78 ± 3.93, n = 9 and bal: 0.55 ± 0.93, n = 11). (B-D) Statistical analysis of atrial cardiomyocyte numbers (wt: 97.29 ± 11.54; bal: 90.43 ± 7.48, n = 7), EdU+ cardiomyocytes in the atrium (wt: 3.71 ± 1.38, bal: 2.00 ± 1.53, n = 7) and the proliferative index in the atrium (wt: 3.79 ± 1,22%, bal: 2.22 ± 1.65%, n = 7). Error bars indicate s.d., ***p < 0.001, ns, not significant. (TIF) [file pgen.1009890.s001.tif]

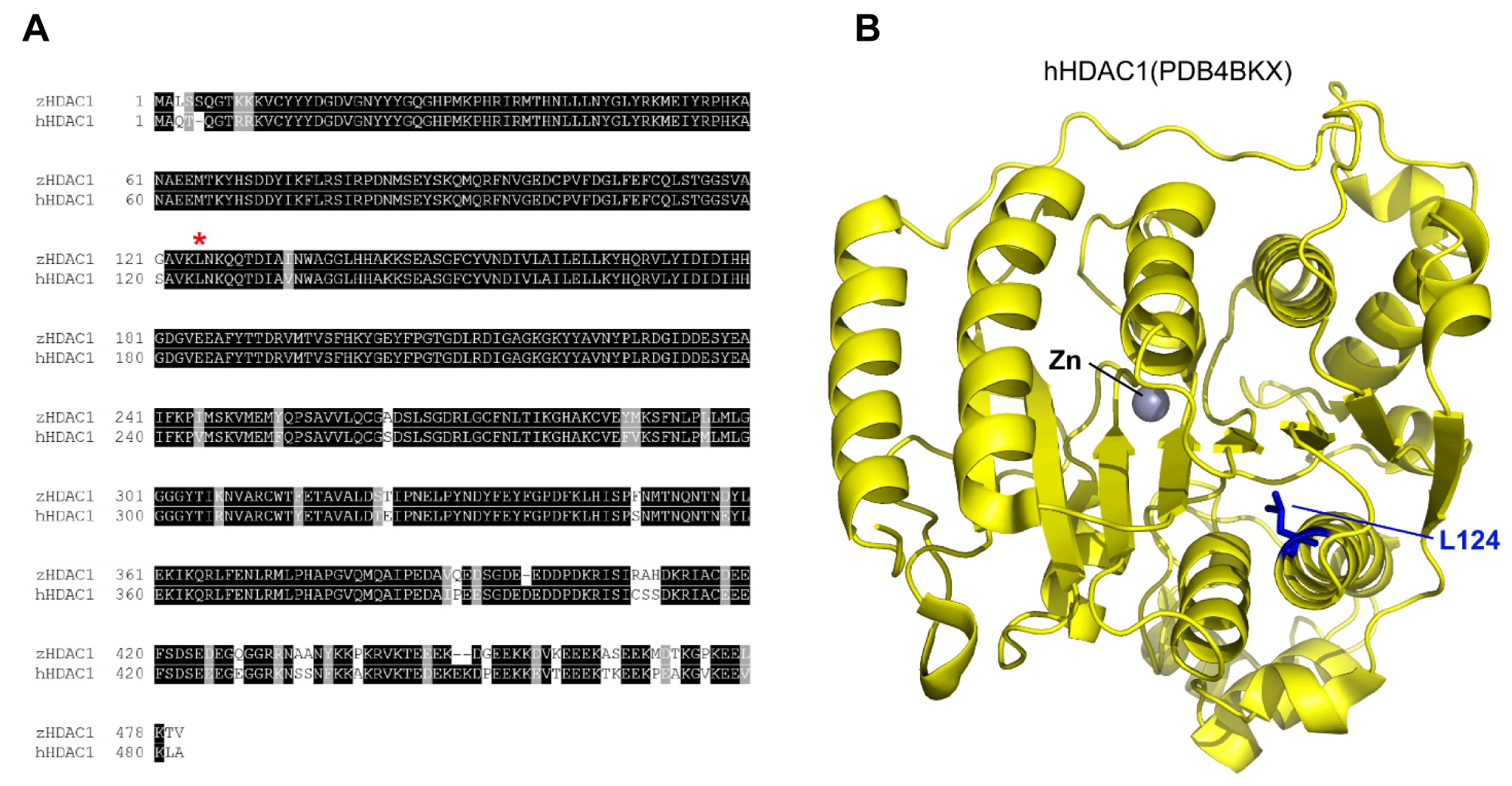

Supplement: S2 Fig — (A) Amino acid (aa) alignment of zHdac1 and hHDAC1 depicts high amino acid identity across species. Identical aa in black, similar aa in grey and no similarity in white. The red box and the asterisk indicate the bal mutation at position 125 of zebrafish Hdac1. (B) Overall structure of human HDAC1 (PDB4BKX, [22]) highlighting its secondary structure elements as cartoon presentation. The zinc ion (Zn) in the active center is shown as grey sphere. The leucine residue at position124 is shown as stick presentation (blue). (TIF) [file pgen.1009890.s002.tif]

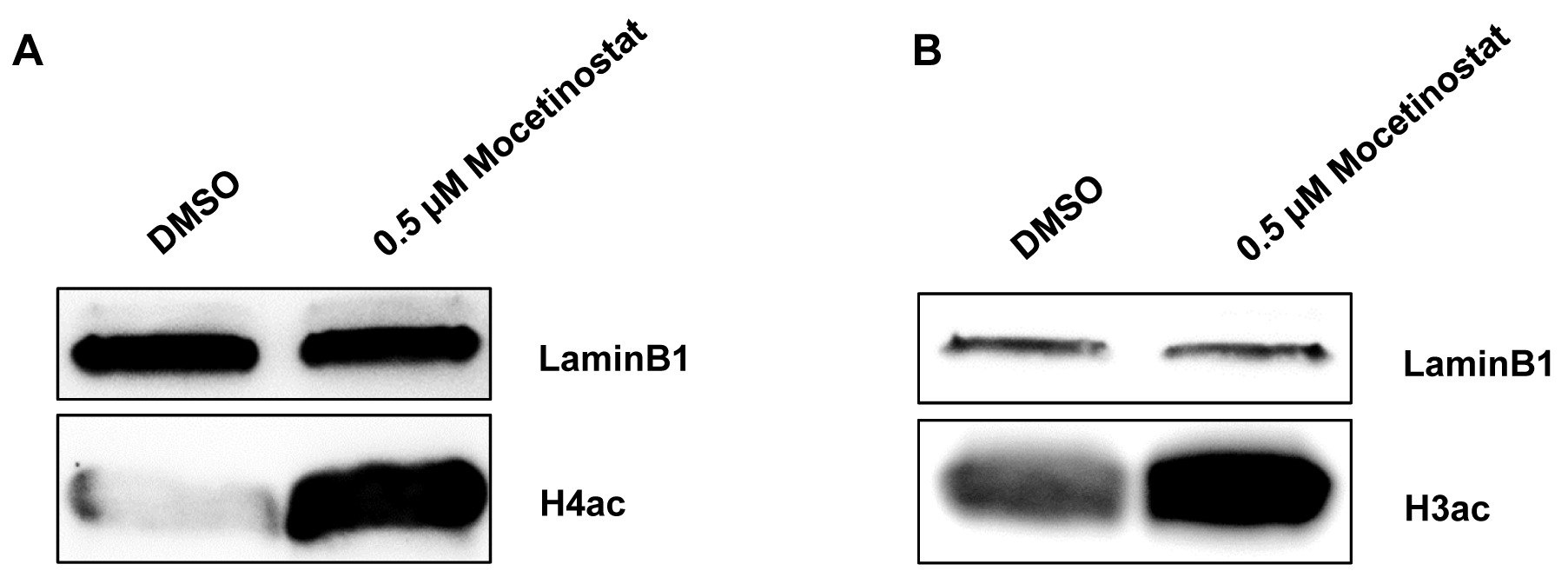

Supplement: S3 Fig — (A, B) Western blot analysis of acetylation levels of Histone 3 (H3ac) and 4 (H4ac) of lysates of ventricles derived from Mocetinostat- and DMSO-treated fish show a pronounced hyperacetylation of H3 and H4 in inhibitor treated ventricles. LaminB1 served as loading control. (TIF) [file pgen.1009890.s003.tif]
